# Targeting the MR1-MAIT Cell Axis for Vaccination Against Infectious Disease

**DOI:** 10.3390/vaccines14020117

**Published:** 2026-01-26

**Authors:** Mattie S. M. Timmer, Lisa M. Connor, Bridget L. Stocker

**Affiliations:** 1 School of Chemical and Physical Sciences, Victoria University of Wellington, Wellington 6012, New Zealand; mattie.timmer@vuw.ac.nz; 2 Centre for Biodiscovery, Victoria University of Wellington, Wellington 6012, New Zealand; 3 Malaghan Institute of Medical Research, Wellington 6242, New Zealand

**Keywords:** MAIT cell, MR1, 5-OP-RU, ligand, immune cell, vaccine, adjuvant, infectious disease

## Abstract

Mucosal-associated invariant T (MAIT) cells exist in high numbers in the body and have a unique and highly conserved T cell receptor (TCR). They can be activated in a TCR-dependent manner by ligands presented on the monomorphic protein MHC class I-related protein 1 (MR1) which is found on many cell types, including professional antigen presenting cells (APCs) and epithelial cells. This has sparked interest in the potential to exploit the MR1-MAIT cell axis for the development of vaccines against infectious disease. Within this context an MR1 ligand, typically 5-(2-oxopropylideneamino)-d-ribitylaminouracil (5-OP-RU), is administered with or without a Toll-like receptor (TLR) ligand or cytokine in a pan vaccination approach that would prime the immune response to provide protection against a variety of bacterial and viral pathogens. This strategy has led to enhanced protection in murine models of *Legionella longbeachae*, *Francisella tularensis*, *Klebsiella pneumoniae*, *Streptococcus pneumoniae* and influenza infection. However, studies against *Mycobacterium tuberculosis* infection have proven less successful. The second vaccination approach involves pairing the MR1 ligand with more conventional antigens that could activate CD4^+^ and/or CD8^+^ T cells. This approach has been successful in murine models of cholera, influenza, and SARS-CoV-2, including in the context of subunit vaccines. However, there are several challenges when using MR1-MAIT cell-mediated vaccine adjuvants. These include the inherent instability of 5-OP-RU and the need for more advanced studies to better understand how the use of MR1 ligands would translate to applications in humans. This review will discuss these aspects and highlight the mechanistic studies that have been undertaken to understand how MAIT cells might elicit their effects within the context of MAIT cell-mediated vaccines for infectious disease.

## 1. MAIT Cells and Infectious Disease

Mucosal-associated invariant T (MAIT) cells were first detected in 1993 by Porcelli and co-workers [1]. Since this time, there has been growing interest in the role of MAIT cells in medicinal applications, including in the development of vaccines [2]. MAIT cells are present in high numbers in the body (up to 10% of circulating human T cells, and up to 45% in the liver) [3,4], and, as their name suggests, they can be found in mucosal sites including the respiratory, oral, intestinal, and urogenital tracts, and in the skin [5]. Accordingly, MAIT cells are in frequent contact with the environment and the microbiota of the host and are thought to play a critical role in safeguarding the mucosa against external microbial threats [5,6,7]. Unlike conventional T cells, which are heterogeneous and are activated by ligands for the polymorphic major histocompatibility complex (MHC) molecules MHCI and MHCII, MAIT cells express a unique and highly conserved T cell receptor (TCR) [7,8]. The α chain of MAIT cells in humans is restricted to Vα7.2-Jα33/12/20, while in mice this is Vα19-Jα33 [8], with preferential use of Vβ2 and Vβ13 β chains in humans and Vβ6 and Vβ8 in mice [8]. Because MR1 is non-polymorphic and MAIT TCRs are conserved, MAIT cells can be targeted in a broad population, making them attractive targets for vaccine development.

**Figure 1:**
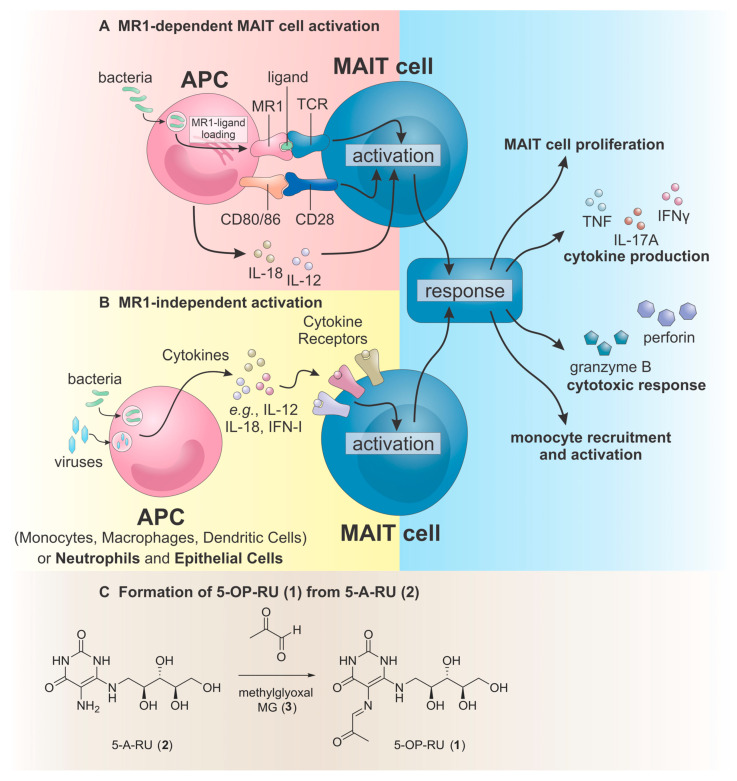
(**A**) MR1-dependent MAIT cell activation. TCR-dependent MAIT cell activation involving MR1-mediated presentation of antigen. MAIT cell co-stimulation can occur by CD28-CD80/86 interactions and by cytokines from the APC in response to the pathogen, including the inflammatory cytokine response to TLR ligands. (**B**) MR1-independent MAIT cell activation. TCR-independent (cytokine-dependent) MAIT cell activation requires the action of multiple cytokines, which can be generated in response to the phagocytosis of bacteria and/or viruses, and TLR ligands. (**C**) Formation of the prototypical MR1-dependent MAIT cell agonist, 5-OP-RU (**1**), occurs in situ from the reaction of 5-A-RU (**2**), an intermediate in the riboflavin biosynthesis pathway, and methylglyoxal (MG, **3**), a by-product of glycolysis.

MAIT cells have evolved to recognise non-proteinogenic microbial antigens using a similar process to that of conventional T cells [6,7,9]. The TCR-dependent MAIT cell activation process relies on the presentation of MAIT TCR ligands by a monomorphic protein, MHC class I-related protein 1 (MR1), which is expressed on a variety of cells, including antigen presenting cells (APCs) [3,4,5] (Figure 1A). MR1 remains inside the endoplasmic reticulum in an immature form until it encounters the appropriate ligand [e.g., 5-(2-oxopropylideneamino)-d-ribitylaminouracil (5-OP-RU), **1**, Figure 1C]. At this point, a covalent bond between Lys43 on MR1 and the ligand is formed and the endoplasmic MR1 is refolded into the active conformer and expressed on the surface of the APC [10,11]. 5-OP-RU is the prototypical TCR-dependent MAIT cell agonist and is formed by a non-enzymatic chemical reaction between 5-amino-d-ribitylaminouracil (5-A-RU, **2**), an intermediate in the riboflavin (vitamin B2) biosynthesis pathway, and methylglyoxal (MG, **3**), a by-product of glycolysis [7,12,13]. Accordingly, many bacteria (e.g., *Escherichia*, *Lactobacillus*, *Staphylococcus*, *Shigella flexneri*, *Salmonella*, *Mycobacteria*, and *Clostridioides*) and fungi (e.g., *Saccharomyces*, *Candida*, *Aspergillus*) that express the riboflavin pathway can activate MAIT cells [9].

TCR-dependent MAIT cell activation is nuanced and exhibits great diversity depending on the disease or illness in question [6]. This is because MAIT cell co-stimulation (e.g., through CD28 and CD40L, by cytokines, or via the effects of Toll-like receptor (TLR) agonists), can affect the MAIT cell response [6,9,14]. In general, TCR-dependent MAIT cell activation leads to the production of inflammatory cytokines (e.g., interferon (IFN)-γ, TNF-α, and interleukin (IL)-17), as well as granzyme B and perforin which can lyse cells [6,7,9,15]. In mice, MAIT cells are categorised into MAIT17 cells, which produce IL-17, or MAIT1 cells, which produce IFN-γ [15,16,17]. Human MAIT cells exhibit a more uniform phenotype combining both Th1 and Th17 characteristics with co-expression of T-bet^+^ (MAIT1) and RORγt^+^ (MAIT17), although the MAIT1 phenotype predominantly dominates [15,16,17]. The development of MAIT17 cells is thought to occur during certain illnesses, such as pneumonia [18], or during chronic autoimmune, metabolic, or liver disease [6].

In addition to TCR-dependent MAIT cell activation, MAIT cells can be activated in a TCR-independent (cytokine-dependent) manner (Figure 1B) [6,7,9,15]. This alternative mode of activation broadens the potential range of pathogens that MAIT cells can respond to and allows them to respond to viral infections. MAIT cells express many cytokine receptors that detect cytokines such as IL-7, IL-12, IL-15, IL-18 and type-I interferons (IFN-I), although TCR-independent MAIT cell activation typically requires synergistic cytokines (e.g., IL-12 and IL-18) to mediate a MAIT cell response [6,7,9,15,19]. It is thought that this multi-pronged cytokine requirement is present to limit what could otherwise be chronic MAIT cell activation and associated immunopathologies [5].

The ability of MAIT cells to be activated by TCR-dependent and independent mechanisms and the presence of MAIT cells in mucosal tissue has seen them play an important role in the host defence against a range of viral (e.g., dengue virus, hepatitis C virus, influenza virus [20], and COVID-19 [21]) and bacterial (e.g., *Legionella* [22], *Francisella* [23,24,25] and *Klebsiella pneunoniae* [26]) infections. Some fungal pathogens that synthesise riboflavin, including *Candida*, *Aspergillus* and *Mucorales* species, can also activate MAIT cells in a TCR-dependent manner [27,28,29], although the protective effect of MAIT cells in this context is less well studied. The MAIT cell effector function is tuneable and the role of MAIT cells during infection is complex and can change depending on disease progression [6]. This is an intricate topic, and the reader is directed towards several recent reviews on the role of MAIT cells in disease [30,31,32,33,34,35]. The adjuvant potential of MAIT cells that are activated in a bystander manner by vaccine-induced responses has also been reviewed elsewhere [2,15,30,35]. Herein, we will focus on TCR-dependent MAIT cell activation via the agency of exogenously added MAIT cell antigens as a strategy for the development of vaccines against infectious disease. The therapeutic use of 5-OP-RU in the context of *M. tuberculosis* infection is also discussed as this brings some context to other prophylactic vaccination studies.

## 2. TCR-Dependent MAIT Cell Activation for Vaccination Against Pathogens

**Figure 2:**
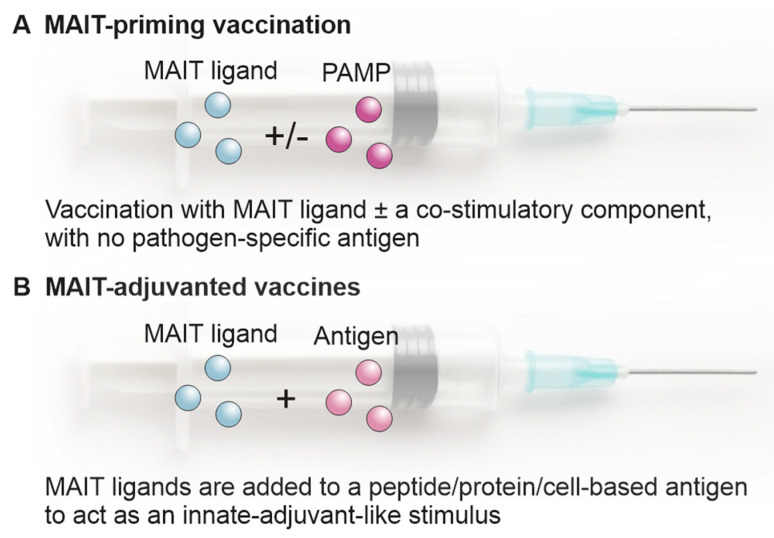
(**A**) MAIT cell-mediated vaccine platforms involving the use of MAIT cell antigens with or without TLR ligands. (**B**) Alternatively, vaccination studies can be undertaken using MAIT cell antigens and conventional peptide/protein antigens.

There are two general TCR-dependent MAIT cell-mediated therapies that can be used to provide protection against infectious disease (Figure 2). The first is a pan-vaccination approach involving vaccination with a MAIT cell antigen (e.g., 5-OP-RU) but no further antigen (Figure 2A). Here, priming of MAIT cells can generate a population of MAIT cells with memory-like recall properties so that expanded MAIT cells persist in a poised effector state and respond rapidly to subsequent infectious challenge. In vivo, antigen-driven MAIT activation/accumulation is strongly shaped by co-stimulatory and inflammatory cues and can result in robust effector outputs (e.g., IFNγ, TNF, IL-17) that support antimicrobial defence and promote recruitment/activation of other innate effectors [36]. Prophylactic MAIT agonism has been reported to reduce disease severity in several models (Table 1) via potential mechanisms that involve the combination of heightened early effector function and immunomodulatory effects that tune myeloid cell activity and dampen immune inflammation (e.g., regulation of neutrophil/macrophages antimicrobial programmes in pneumococcal pneumonia) [37]. In addition to 5-OP-RU, TLR agonists or cytokines are often included in the vaccine formulation [36,38].

However, the durability of these memory-like MAIT responses remains incompletely defined. While antigen-driven MAIT expansion can persist for weeks, it is not yet clear that MAIT memory endures for months to years as classical adaptive memory dose. This limited or uncertain longevity raises the possibility that a pan-vaccination strategy based solely on MAIT agonists may provide a relatively short therapeutic window and therefore be most useful for short-term prophylaxis in outbreak or pandemic settings rather than as a long-lasting vaccine.

The second, more conventional, vaccination strategy involves the use of MAIT cell antigens in combination with cell-based antigens that would be processed by APCs and presented as peptide fragments on MHC-I (for endogenous antigens) or MHC-II (for exogenous antigens) to conventional CD4^+^ T or CD8^+^ T cells, respectively [39] (Figure 2B). In this context, the MAIT cell antigen would act as an adjuvant in the classical sense of the word by enhancing the immune response to the peptide antigen. Carbohydrate-based vaccines, which rely on the generation of antibodies against polysaccharides found on the cell surface of the pathogen [40], represent another vaccination strategy that might be augmented via the addition of MR1-mediated MAIT cell activating ligands.

### Vaccination Against Infection Using MR1-Mediated Adjuvants

Most MAIT TCR-mediated vaccinations have used 5-OP-RU as the activating ligand. This ligand, along with 5-(2-oxoethylideneamino)-6-d-ribitylaminouracil (5-OE-RU), which is formed via the condensation of 5-A-RU and the glycolysis pathway by-product glyoxal, was discovered during mutagenesis experiments to investigate the genes involved in the bacterial riboflavin metabolism pathway [12]. 5-OP-RU was found to bind to MR1 via the formation of a covalent bond (Schiff base) with Lys43 in the MR1 binding pocket [12,41]. The ribityl group of 5-OP-RU can then engage with the MAIT cell TCR. 5-OP-RU is an exquisitely potent MAIT cell activator with an EC_50_ = 0.1–5 nM, depending on the assay, however it is also inherently unstable (t_1/2_ = 88 min, PBS, pH 7.4, 37  °C) [41] as it is prone to cyclisation, to form the lumazine scaffold, and hydrolysis of its imine functionality [12,41].

**Table 1:** MR1 Antigen (no protein antigen).

MR1 Ligand/ Antigen	Disease Model	MAIT Cell Expansion	MAIT Cell Phenotype	Mechanistic Insight	Challenge	Reference
5-OP-RU	*Salmonella* (BRD509Δ*ribDH*)	No	MAIT CD69 ↑	5-OP-RU alone could not expand MAIT cells	_	Chen et al. 2017 [36]
5-OP-RU + Pam_2_Cys 5-OP-RU + CpG 5-OP-RU + poly I:C	*Salmonella* (BRD509Δ*ribDH*)	Yes	MAIT CD69 ↑	MAIT cells redundant in protection against *S. Typhimurium* (BRD509Δ*ribDH*)	_	Chen et al. 2017 [36]
5-OP-RU + CpG 5-OP-RU + Pam_2_Lys	*L. longbeache* (murine)	_	_	MAIT cell response to bacteria only (no 5-OP-RU) was INF-γ/GM-CSF dependent	CFU ↓ (lung)	Wang et al. 2019 [42]
5-OP-RU + IL-23	*L. longbeache* (murine)	Yes	MAIT17 dominates	MAIT activity ICOS on MAIT cells	CFU ↓ (lung)	Wang et al. 2019 [42]
5-OP-RU + CpG	*L. longbeache* (murine)	Yes (prior to infection)	Mainly MAIT1 (prior to infection)	Effect abrogated in MR1^−/−^ mice	CFU ↓ (lung)	Zhao et al. 2018 [24]
5-OP-RU + CpG	*F. tularensis* (murine)	Yes (prior to infection)	Mainly MAIT1 (prior to infection)	Effect abrogated in MR1^−/−^ mice	CFU ↓ (liver, lung) 60% survival (lethal otherwise)	Zhao et al. 2018 [24]
5-OP-RU + LPS	*K. pneumoniae* (murine)	Yes	MAIT CD69 ↑	Cytokine profile (no change), macrophage and neutrophil lung infiltration ↓, Type I IFN dependent	CFU ↓ (lung) less weight loss inflammation ↓	López-Rodríguez et al. 2018 [43]
5-OP-RU + Pam_2_Cys	*M. tuberculosis* (murine)	Yes, then No	MAIT CD69 ↑ then ↓	MAIT response decreased over time	No change CFU (lung), no increase in survival	Vorkas et al. 2020 [44]
5-OP-RU + CpG	*M. tuberculosis* (murine)	Yes: prior to infection	MAIT17 dominates prior to infection	Delayed *M.Tb* CD4^+^ T cell priming post challenge; partially TGF-β dependent	No change CFU (lung) CFU ↓ in mLN	Sakai et al. 2021 [45]
5-OP-RU	*S. pneumoniae* (murine)	No: prior to infection	Dose-dependent response (CD69 ↑, PD-1 ↑, IL-17A ↑) prior to infection	Neutrophils ↑ (prior to infection)	CFU ↓ (lung) Enhance survival (75% *c.f.* lethal infection)	Barsac et al. 2025 [37]
5-OP-RU	Influenza A (murine)	_	_	_	100% survival	Pankhurst et al. 2023 [46]
5-OP-RU + Pam_2_CSK_4_ (Therapeutic use)	*M. bovis* (murine)	Yes	MAIT17 dominates	MAIT cells inhibit BCG growth in BMDM in vitro	CFU ↓ (lung)	Yu et al. 2020 [47]
5-OP-RU + Pam_2_CSK_4_ (Therapeutic use)	*M. tuberculosis* *BCG* (murine)	Yes	MAIT17 dominates	MAIT cells do not inhibit *M.Tb* growth in BMDM in vitro	No change CFU (lung)	Yu et al. 2020 [47]
5-OP-RU (Therapeutic use)	*M. tuberculosis* (NHP)	No	MAIT Ki-67 ↑	MAIT cell cytokine/granzyme production: no change MAIT PD-1 ↑ MAIT dysfunction upon restimulation	Bacterial loads inconclusive 40% survival cf. 100% (control) No change in CD4^+^/CD8^+^ T cells or IgG	Sakai et al. 2021 [48]
5-OP-RU + CpG (Therapeutic use)	Healthy NHP model	No	MAIT Ki-67 ↑ (BAL, blood, but not lungs)	PD-1 partially responsible for loss of MAIT cell function but not expansion	N/A	Sakai et al. 2021 [48]

## 3. Vaccination with MR1 Ligand Only (No Other Antigen)

### 3.1. 5-OP-RU in the Absence of Proteinaceous Antigens for Antibacterial Vaccinations

Several studies have demonstrated that MAIT cells play a critical role in controlling pulmonary or gastrointestinal bacterial infections [15,31,32,33]. The co-stimulation of MAIT cells by cytokines can also affect their activation status [6,9,14]. With this in mind, MR1-ligands, either alone, or in combination with PAMPs, have been investigated for their ability to provide protection against bacterial infection. The co-administration of 5-OP-RU and cytokines has also been explored.

Early studies into the potential of 5-OP-RU to protect against bacterial infection used *Salmonella* disease models [36]. Here, a mutant strain of *S. Typhimurium* BRD509 with deleted *ribD* and *ribH* genes (BRD509Δ*ribDH*), and which thus lacks an intact microbial riboflavin pathway, was used to infect mice and induce disease.

Intranasal administration of 5-OP-RU along with Pam_2_Cys (TLR2/6), CpG (TLR9), or poly I:C (TLR3) agonists led to a 15–25-fold enrichment of MAIT cells in the lung, whereas the addition of 5-OP-RU alone was insufficient to drive MAIT cell expansion, suggesting that MAIT cell accumulation and enrichment require stronger signals than those needed for activation. However, lung clearance of *Salmonella* was independent of the presence of MAIT cells, which may be redundant as protective T cells in this model.

This work was followed shortly thereafter by Wang et al. who demonstrated that the administration of 5-OP-RU and the TLR ligands CpG or Pam_2_Cys provided protection against pulmonary *L. longbeachae* infection in a MAIT cell-dependent manner [42]. In this work, 5-OP-RU/TLR ligand were administered 4 weeks before challenge with *L. longbeachae* with protection being observed by way of a significant reduction in the bacterial load in C57BL/6 versus MR1^−/−^ mice at 5, 7 and 10 days post infection. Within the context of these studies, the authors also determined that MAIT cells play a significant role in the pulmonary host defence against *L. longbeachae*, with this MAIT-cell-mediated protection being dependent on IFN-γ and being enhanced by granulocyte-macrophage colony-stimulating factor (GM-CSF) [42].

Wang et al. also determined that vaccination with interleukin (IL)-23 plus 5-OP-RU prior to intranasal *L. longbeachae* challenge augments MAIT cell-mediated control of pulmonary Legionella infection, as evidenced by significant decreases in the bacterial load in the lung for the 5-OP-RU/IL-23 treatment group compared to the IL-23 or untreated controls in the week following the challenge [42]. Mechanistically, it was determined that optimal in vivo MAIT cell activation required signalling through the inducible T cell co-stimulator (ICOS), with 5-OP-RU/IL-23 co-stimulation leading to an expansion of a MAIT cell population that was indicative of a MAIT 17 phenotype (RORγt^+^T-bet^+^). Zhao et al. further demonstrated the potential of MAIT cell-targeted vaccination against *L. longbeachae* infection using 5-OP-RU and CpG and extended the approach to *F. tularensis* infection models [24]. In these studies, vaccinated WT C57BL/6 mice exhibited a significant reduction in the bacterial load in the liver and lungs, at 3 and 5 days post infection (dpi) compared to unvaccinated mice, CpG-only treated mice, or vaccinated *Mr1*^−/−^ mice. Mice vaccinated with 5-OP-RU and CpG also showed enhanced survival (60% at 30 dpi) following otherwise lethal *F. tularensis* infection [24]. The majority of MAIT cells boosted with this vaccination scheme had a MAIT-1 phenotype.

In 2023, Barral and co-workers published findings that demonstrated the role of type I interferons in providing protection against *K. pneumoniae* as a model of bacterial pneumonia [43]. Activation of MAIT cells during infection with *K. pneumoniae* was found to be independent of MR1 and to be primarily driven by type 1 IFN signalling, which is consistent with cytokine-mediated MAIT activation during bacterial infection. Because baseline levels of MAIT cells are low in pathogen-free C57BL/6 mice, Barral and co-workers then considered whether pre-expanding/priming MAIT cells with MR1 ligands in combination with a TLR stimulus could improve host defence against subsequent *K. pneumoniae* infection. To this end, WT mice were treated with 5-OP-RU + LPS, PBS (vehicle) or LPS, and were then infected with *K. pneumoniae* one week later. The 5-OP-RU + LPS regimen induced a strong increase in the total MAIT cell number and increased CD69 expression relative to control groups, whereas broader cytokines and transcription factor profiles were not substantially different at the time points assessed. Remarkably, the administration of 5-OP-RU + LPS conferred protection from *Klebsiella* infection, as evidenced by a decrease in bacterial burden and lung inflammation, and the prevention of weight loss in vaccinated animals. These findings were accompanied by a decrease in macrophage and neutrophil recruitment into the lung, which was thought to be the cause of the lung inflammation in the control group. In contrast, LPS treatment alone led to poorer outcomes with increased CFUs in the lung, worse lung injury, and greater weight loss. To probe the mechanism of this MAIT-associated protection, mice were injected with blocking antibodies to IFNAR (type I IFN receptor) or MR1 after MAIT priming (i.e., 3 days after the last 5-OP-RU/LPS treatment), but before bacterial challenge [43]. Under these conditions, protection remained independent of MR1at the time of infection but was abrogated by IFNAR blockade, indicating that type I IFN signalling is required for the protective programme elicited by MAIT priming. Further studies would be required to tease apart the mechanism by which type I IFN mediates protection by MAIT cells (e.g., via MAIT effector functions, and/or downstream modulation of myeloid inflammation) during *Klebsiella* infection.

Despite promising results for MAIT cell priming in several bacterial infection models, MAIT cell enrichment with 5-OP-RU to provide protection against *M. tuberculosis* infection has been met with less success. In initial murine models, mice were primed via intranasal inoculation with 5-OP-RU and Pam_2_Cys (TRL 2/6 agonist) and infected with 50 to 150 CFU/lung of *M. tuberculosis* via aerosol 14 days after initiation of priming [44]. This led to robust early MAIT cell activation and expansion in the lung after *M. tuberculosis* challenge. However, 5-OP-RU/Pam_2_Cys priming did not translate into improved bacterial control, with no impact on bacterial load compared with control groups. Importantly, priming also did not measurably enhance the subsequent *M. tuberculosis*-specific immune response, including TB10.4-specific CD8^+^ T cell and ESAT6-specific CD4^+^ T cell populations, nor did priming lead to expansion of other immune subsets (e.g., γδ T, B, NK or myeloid cells), consistent with the conclusion that MAIT enrichment did not “boost” the conventional response that develops after infection. Strikingly, despite significant recruitment of MAIT cells to the lungs after bacterial exposure, these cells were depleted from the lung by the time the adaptive immune response emerged, with reduced detection of granzyme B^+^ and IFN-γ^+^ MAIT cells compared with uninfected Pam_2_Cys/5-OP-RU treated mice. A series of follow-up approaches, including lowering the infectious dose, altering the interval between priming and infection, and testing in NOS2-deficient mice, failed to reveal any protective effect of Pam_2_Cys/5-OP-RU on *M. tuberculosis* growth [44].

Similar findings were observed by Sakai et al. in another prophylactic murine *M. tuberculosis* vaccination study using 5-OP-RU + CpG, which were administered by intrapharyngeal inoculation [45]. In preliminary studies, 5-OP-RU + CpG, but not 5-OP-RU alone, led to the expansion of MAIT cells in the lung. The combination of 5-OP-RU + CpG was then used in vaccination studies. When mice were inoculated with 5-OP-RU + CpG six days before *M. tuberculosis* aerosol challenge, vaccination did not lead to a decrease in bacterial loads in the lung, but rather was associated with delayed *M. tuberculosis*-specific CD4^+^ T cell priming in the lung-draining lymph nodes via a mechanism that is partially dependent on transforming growth factor (TGF)-β. Consistent with a delay in the early seeding events that initiate priming, vaccinated mice showed reduced bacterial loads in the mediastinal lymph nodes (mLNs). The authors note that transport of *M. tuberculosis* to the mLN and the subsequent priming of adaptive immunity depends on the trafficking of CCR2^+^Ly6C^+^ monocytes to this site [49]. MAIT cell vaccine with 5-OP-RU + CpG markedly reduced CCR2^+^Ly6C^+^ myeloid cells in the mLN, and TGF-β blockade partially restored these cells in vaccinated mice. Notwithstanding, Sakai et al. also performed murine experiments that gave some initial optimism that 5-OP-RU could be used as a therapy for established tuberculosis [45]. These studies are described in Section 3.3 along with other therapeutic uses of 5-OP-RU for *M. tuberculosis* infection.

Finally, within the context of 5-OP-RU-mediated prophylactic vaccines, intranasal treatment with 5-OP-RU alone has been shown to protect mice from pneumococcus-induced lethal pneumonia [37]. At the time of writing, this work is yet to be peer-reviewed. In these studies, it was determined that, prior to *Streptococcus pneumoniae* infection, the administration of 5-OP-RU led to a decrease in MAIT cell frequency, an increase in MAIT cells expressing CD69 and PD-1 and producing IL-17A in a dose-dependent manner (0.02–0.2 nM), with higher doses of 5-OP-RU leading to the greater increase in these immune responses. No change in MAIT Ki-67 expression was observed, which suggests no early proliferation of the MAIT cells. It was then determined that a low dose (0.2 nM) of 5-OP-RU led to an increased number of neutrophils in the lung of treated mice, and, importantly, that the intranasal administration of 5-OP-RU prior to *S. pneumoniae* challenge led to reduced bacterial burdens in the lungs and limited systemic dissemination of the bacteria. WT mice treated with 5-OP-RU also had enhanced survival (75% at day 8), while all other treatment groups, including the WT control group and MR1^−/−^ mice, succumb to infection on day 5. In addition, lung MAIT cells from 5-OP-RU treated mice could respond to a secondary challenge with 5-OP-RU. This was in the absence of a bacterial challenge but is still important to note given that other studies have demonstrated MAIT cell anergy following stimulation with 5-OP-RU.

### 3.2. 5-OP-RU in the Absence of Proteinaceous Antigens Viral Vaccinations

To date, only one study has been conducted on anti-viral vaccination using only a MAIT cell antigen. Here, Pankhurst et al. investigated the ability of 5-A-RU/MG to provide protection against influenza A challenge [46]. Mice were treated three times with 5-A-RU/MG over a three-week period and then challenged three weeks later with X31 influenza A virus. Prior MAIT cell priming with 5-A-RU/MG conferred a clear survival benefit to influenza infection with all animals surviving viral challenge. However, in the same report, they also tested MAIT cell activation as a cellular adjuvant by co-administering influenza A protein hemagglutinin (HA) together with 5-A-RU/MG. In this setting, the mice recovered from infection at a faster rate, as evidenced by a rapid regaining of starting weight relative to the 5A-RU/MG only group (also see Section 4.3). This is the first study to compare the effect of MAIT antigen +/− protein antigen. It would be valuable to determine whether other studies lead to similar findings and support the concept that MAIT cells can shape downstream adaptive immunity.

### 3.3. 5-OP-RU for the Treatment of M. tuberculosis Infection (Therapeutic Context)

Although the results for the effectiveness of 5-OP-RU as a prophylactic vaccine for *M. tuberculosis* were discouraging, MAIT cell stimulation with 5-OP-RU post *M. tuberculosis* infection in mice led to an approximate 1 log reduction in bacterial loads in the lung and was found to be MR1-dependent [45]. The use of 5-OP-RU in a therapeutic context is outside the main scope of this review. However, because studies within the context of *M. tuberculosis* infection provide important insight into the relevance of prophylactic vaccines using 5-OP-RU in non-human primate (NHP) models, this work is described below.

In murine studies, 5-OP-RU and the TLR2/6 agonist, Pam_2_CSK_4_, led to MAIT cell recruitment and activation in the lung for both *M. Bovis BCG*- and *M. tuberculosis*-infected mice [47]. However, only *M. Bovis* BCG growth, but not *M. tuberculosis* growth, was effectively contained. Surprisingly, the cytokine profile of the induced MAIT cells harvested from the lungs of *BCG* or *M. tuberculosis*-infected mice was similar with high levels of IL-17A and very low, but detectable, levels of IFN-γ and TNF being observed on day 7 post infection. This data suggests that 5-OP-RU/Pam_2_CSK_4_ can lead to the accumulation of functionally competent MAIT cells in the lung of *M. tuberculosis*-infected mice, but these cells lack the capacity to inhibit *M. tuberculosis* growth in vivo. Several theories have been proposed to account for this including the production of IL-10 by *M. tuberculosis*-infected macrophages, which would inhibit the antibacterial activity of macrophages [47], or the production of inhibitory MR1 ligands by the bacteria that would then suppress MAIT cell activation [44,45].

Because of the difference between the MAIT cell populations in humans and mice, both in terms of phenotype and number (higher number of MAIT cells in humans), Sakai et al. deemed it worthwhile to explore the therapeutic potential of 5-OP-RU in a NHP model of *M. tuberculosis* [48]. To this end, *M. tuberculosis*-infected rhesus macaques were treated with 5-OP-RU during weeks 6–14 post infection. This treatment regime led to MAIT cell activation but no MAIT cell expansion or enhancement in cytokine and granzyme production. However, upregulation of the marker for programmed death-1 (PD-1) on MAIT cells was observed, which is typically associated with T cell activation, and in some contexts, T cell exhaustion. 5-OP-RU also led to MAIT cell dysfunction in the *M. tuberculosis*-infected macaques, as evidenced by the inability of the MAIT cells in the treated animals to respond to restimulation with 5-OP-RU. There was also no effect following 5-OP-RU treatment on *M. tuberculosis*-specific CD4^+^ T cell, CD8^+^ T cell, or IgG responses. In addition, of the five macaques treated with 5-OP-RU, three needed to be euthanised early (weeks 7, 10 and 12), while all five macaques were alive in the PBS group at week 14. Bronchoconstriction was observed in 2/5 macaques that were euthanized, which was attributed to enlarged lymph nodes constricting the airways. However, the authors suggest interpreting this result with caution due to the small sample size, the tendency of rhesus macaques to develop TB lymphadenopathy, and there being no obvious biological effect of the MAIT cells that could be ascribed the lymph node disease.

To understand more about the rationale for the lack of MAIT cell expansion in the rhesus macaques, Sakai et al. conducted a further study in uninfected animals using a lower (ten-fold decrease) dose of 5-OP-RU that was co-administered with CpG [48]. For some groups, PD-1 blockade was also implemented. The MAIT cells also failed to expand in the airways after 5-OP-RU/CpG treatment, although Ki-67 was significantly upregulated in the bronchoalveolar lavage (BAL) and blood. PD-1 blockade had no effect on MAIT cell frequency but may have slightly reduced Ki-67 expression in the BAL. Taken together, these findings bring into question whether prophylactic or therapeutic 5-OP-RU-mediated MAIT cell therapies are viable approaches for the prevention or treatment of *M. tuberculosis* [50], or indeed, for any disease given the failure of MAIT cell expansion in NHP models. Investigations into the use of other MR1 ligands or treatment regimes, such as dosage, would be required to explore this question. As part of a broader approach, it should also be noted that in NHPs vaccinated with BCG, the MAIT cells were activated and were found to respond quickly to *M. tuberculosis* infection [51]. This suggests that MAIT cell-mediated strategies may still be relevant in the development of vaccines for *M. tuberculosis* but may point to the need for studies involving the use of 5-OP-RU in combination with existing TB vaccination platforms (i.e., using an approach more akin to those described in Section 4). It is also interesting to note that individuals who are resistant to *M. tuberculosis* infection have increased frequencies of MR1-restricted T (MR1T) cells with a relatively diverse TCR repertoire [52]. MR1T cells lack the conserved TCR gene usage that characterises MAIT cells and may recognise alternative ligands [53]. Although outside the remit of “MAIT” cells, it would be valuable to determine whether there are specific ligands that can preferentially bind to the TCR of MR1T cells and elicit bacterial clearance.

## 4. Vaccination with MR1 Antigen and Other CD4^+^ or CD8^+^ T Cell Antigens

### 4.1. MR1 Ligand in Combination Cognate Antigens (e.g., OVA)

Compared to studies investigating the effect of 5-OP-RU (and TLR ligands) that solely rely on the generation of a memory MAIT phenotype, fewer experiments have investigated the ability 5-OP-RU to act as an adjuvant in more conventional vaccination strategies (Table 2). As part of their mechanism studies into the mode of action of viral vaccination platforms for influenza and SARS-CoV-2 (Section 4.3), Pankhurst et al. investigated the effects of 5-A-RU/MG on OVA-specific immune cell responses [46]. In this work, mice were given three doses of 5-AR-U/MG + OVA which led to MAIT cell activation and proliferation (peaking at day 3 then returning to baseline by day 5), but minimal inflammatory cytokines. Despite this fleeting rise in MAIT cell number, the effect was sufficient to lead to CD40L-CD40 MAIT-DC signalling and the activation of migratory DCs and OVA-specific T follicular helper (T_FH_) and germinal centre (GC) B cells. OVA-specific IgG and IgA antibody responses were also observed.

To explore the ability of their new TCR-dependent MAIT cell agonist (an aldehyde functionalised ribityllumazine) as a MAIT cell-mediated vaccine adjuvant, Takasaki et al. demonstrated that this compound could enhance the OVA-specific immune response, as determined by a significant enhancement in total IgG, IgG1, IgG2, and IgG2a compared to OVA alone [54]. These results are encouraging and will hopefully prompt others to explore the effects of more stable TCR-dependent MAIT cell agonists as vaccine adjuvants.

### 4.2. MR1 Ligand in Combination with Antibacterial Vaccines

The only study which has considered the combination of 5-OP-RU with bona fide bacterial antigens was that undertaken by Jennsen et al. who focused on the development of carbohydrate vaccines [55]. In 2022, Jensen et al. used an intranasal prime-boost vaccination model with live *V. cholerae* and 5-A-RU + MG as a mucosal adjuvant. Mice were inoculated on day 0 and day 28 with *V. cholerae* O1 Inaba along with either Pam_2_CSK_4_ (Pam_2_, a TLR2/6 agonist), or in combination with 5-A-RU + MG. Despite significant expansion and persistence of mucosal MAIT cells through high- and low-dose intranasal 5-A-RU treatment, the MAIT TCR ligand has limited effects of *V. cholerae*-specific antibody responses and B-cell differentiation, indicating that MAIT activation alone was not sufficient to reliably amplify humoral immunity in this setting.

A further series of experiments was then performed using a low dose of 5-A-RU/MG and the capsular polysaccharide *V. cholerae* O1 Ogawa OSP conjugated to bovine serum albumin [55]. A significantly higher number of MAIT cells were found in the lungs and BAL of these mice, and carbohydrate-specific IgG antibodies were significantly increased in the BAL, but nowhere else. The authors hypothesised that greater IL-21 production by the expanded MAIT cell population in the BAL for the 5-OP-RU/MG treated group might be responsible for the higher antibody titre in the BAL. Notwithstanding, these studies were disappointing as there was little effect on systemic antibody and B-cell differentiation. Thus, while MAIT cells can provide “helper-like” signals and shape the mucosal environment, they do not consistently substitute for conventional CD4^+^ T cell/T_FH_ help [46], which remains critical for robust B cell differentiation and durable systemic antibody responses, particularly for carbohydrate antigens where effective immunity typically depends on protein-containing formulations that can recruit classical T_FH_ responses. As part of their conclusions, the authors noted the challenges of developing carbohydrate-based vaccines in general and suggested that further investigations into dosing, kinetics, or the route of vaccine administration may yield more promising results. The authors also suggested that the development of a *V. cholerae*-LPS *O*-specific polysaccharide:MAIT-ligand conjugate vaccine may be able to improve mucosal IgA responses. Indeed, this is an interesting idea but would warrant the need for a MAIT ligand that is more stable than 5-A-RU, or for a 5-A-RU pro-drug approach, like that developed by Lange et al. [57].

### 4.3. 5-OP-RU in Combination with Proteinaceous Antigen for Viral Vaccinations

The first paper to demonstrate how 5-OP-RU can be used to augment the efficacy of viral vaccines was published in 2023 by Haeryfar and co-workers [56]. In this work, 5-OP-RU was combined with various influenza vaccine platforms or with severe acute respiratory syndrome coronavirus 2 (SARS-CoV-2) vaccine platforms that relied on the use or the recombinant vesicular stomatitis virus to express the Spike protein. In the influenza work, it was determined that the combination of 5-OP-RU and the viral vaccine (A/Puerto Rico/8/1934) led to the in-situ expansion of MAIT cells in multiple tissue and reprogrammed the cells towards a pro-inflammatory (MAIT1 lineage) phenotype. Because MAIT cell activation was not seen in the presence of the heat inactivated virus, the authors hypothesised that a byproduct of viral replication was responsible for promoting MAIT cell activation. Double stranded RNA (dsRNA) is present during viral replication and is sensed by TLR3, triggering type I IFN responses. Given the role for type I IFN in MAIT cell activation [43], this pathway was investigated and it was determined that the ability of the MAIT cells to augment the immune response in the presence of replicating viral vaccines was dependent on the activation of TLR 3 and type I IFN receptor signalling. When using the Flumist vaccine + 5-OP-RU, MAIT cell numbers were also enhanced, as were virus-specific CD8^+^ T cell responses [56].

To explore the protective effects of this vaccination protocol, Haeryfar and co-workers then primed mice i.p. with a non-pathogenic influenza strain [A/Hong Kong/1/1968 (HK) H3N2] and 5-A-RU/MG [56]. The mice exhibited protection against a subsequent heterosubtype pathogenic (PR8) challenge, with 75% survival at 14 days post infection. All surviving mice rapidly regained weight after 5 days. In contrast, all mice died at 5 and 7 days post infection using vehicle (PBS) + HK/H3N2 or 5-A-RU/MG alone, respectively. Vehicle alone (MG in DMSO/PBS) resulted in a 12.5% survival rate at 14 days. It should also be noted that in these studies 5-OP-RU did not lead to MAIT cell anergy, as evidenced by the analysis of the murine MAIT costimulatory/exhaustion markers. MAIT cell proliferation and a proinflammatory immune response were also seen following the treatment of human peripheral blood mononuclear cells (hPBMCs) with an influenza A viral strain and 5-OP-RU [56]. The efficacy of this approach was maintained in young and old mice, which is important to note given that MAIT cell numbers naturally decline with age.

Also in 2023, Pankhurst et al. used 5-A-RU/MG in an influenza A platform using HA protein from the influenza virus [46]. Following an intranasal protocol that involved mice being treated three times with HA + 5-A-RU/MG three weeks prior to challenge, higher levels of protective IgG and IgA antigen-specific antibodies were observed. In an *i.n.* immunisation study using 5-A-RU/MG and HA, 100% protection against live influenza challenge was also observed. This was the first study to demonstrate that 5-OP-RU can be used in combination with subunit vaccines. In contrast, treatment with HA alone, or HA and the commercially available adjuvant, AddaVax, led to more rapid weight loss and 15–20% of the animals did not survive these vaccination regimes. These results hint to an additional protective role of MAIT cells during an inflammatory response, although further work needs to be performed to determine whether priming MAIT cells with 5-A-RU/MG can activate a tissue repair and/or anti-inflammatory programme in this setting. Mechanistic studies involving the use of OVA as a model antigen (as described above) allowed the authors to determine that MAIT cells promoted B cell expansion via their ability to expand CD4^+^ T_FH_ cells, thus offering protection through promotion of adaptive immunity against influenza A.

Further studies by Haeryfar and co-workers [56] and Pankhurst et al. [46] also demonstrated the potential of 5-OP-RU as a MAIT cell-mediated vaccine adjuvant for SARS-CoV-2 vaccines. Haeryfar and co-workers used a recombinant vesicular stomatitis virus (rVSC) to express the Spike protein. Here, *i.m.* priming and boosting with the Spike protein led to a significant increase in pulmonary and splenic MAIT cells [56]. Pankhurst et al. used a subunit vaccine (tandem dimer of the receptor binding domain of the spike protein) [46]. A significant enhancement of antigen-specific IgG and IgA was observed following 5-OP-RU treatment. These antibodies had a neutralising effect.

## 5. The Search for Stable and Potent MAIT Cell Agonists

**Figure 3:**
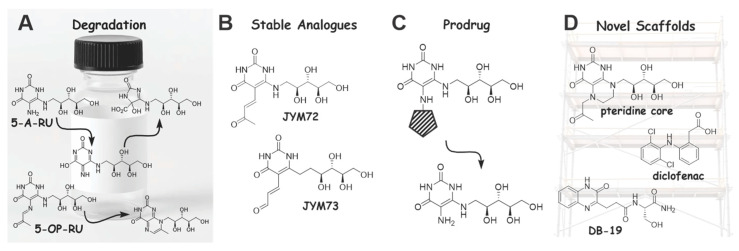
The development of stable and potent MR1-MAIT cell agonists. (**A**) 5-A-RU and 5-OP-RU decompose upon storage. (**B**) Stable analogues in which the labile functional groups have been replaced by chemically more stable ones [61,62]. (**C**) Chemically blocking the amino function in 5-A-RU forms a prodrug that can release 5-A-RU in vivo [57]. (**D**) Novel scaffolds that avoid unstable molecular structures are developed as MR1 ligands [54,59,63].

Despite the potential of 5-OP-RU to augment vaccine efficacy, vaccination studies using this ligand have been relatively limited. This is likely due to the inherent instability of 5-OP-RU, which decomposes to form biologically inactive metabolites, including ribityllumazines [12,41,58] (Figure 3A). The ribtyllumazines cannot upregulate MR1 and exhibit only weak MAIT cell activation [13,58,59]. The challenges with storing and handling 5-OP-RU typically require this ligand to be made directly prior to administration, or being made in situ via the combination of 5-A-RU + MG. However, 5-A-RU also rapidly decomposes and is thought to be readily oxidised by air/light in solution to give an azaquinone, which subsequently undergoes hydrolysis [57]. Accordingly, efforts have been made to develop storage conditions for 5-A-RU that involve the use of DMSO as a solvent [41] or the storage of 5-A-RU as the HCl salt [60]. Although these approaches go a long way in providing better access to 5-A-RU, extending the use of 5-A-RU/5-OP-RU to the clinic remains problematic.

Accordingly, there has been much interest in the development of alternative stable MAIT cell agonists. To date, the development of potent MAIT cell ligands has focused on generating a covalent Schiff base [12] between the compound and MR1 Lys43. This has led to the identification of the 5-OP-RU carbon homologue JYM72 [41,61] and the second-generation derivative thereof JYM73 (Figure 3B) [62], a cathepsin-cleavable pro-drug that delivers 5-OP-RU in situ (Figure 3C) [57], and the previously noted ketone-functionalised ribityllumazine [54] (Figure 3D). It would also be valuable to consider whether the use of potentially higher doses of weaker MAIT agonists (of which there are many, e.g., ribityllumazines, diclofenac derivatives or DB-19) can prevent the MAIT cell exhaustion that has been seen in some vaccination studies using 5-OP-RU. There is certainly much more work that is needed in this area and ready access to sufficiently potent and stable MAIT cell agonists would go a long way in allowing the field to progress. The reader is directed to some excellent recent reviews on the diversity of MR1 ligands [64,65].

## 6. TCR-Dependent MAIT Cell Agonist: Protection Versus MAIT Cell Anergy? Where to from Here?

The ability of MR1-MAIT ligand 5-OP-RU to improve the efficacy of vaccines against infectious disease represents an exciting new opportunity. MAIT cells are quick acting and can not only have a cytotoxic effect but have tissue repair qualities that could help resolve secondary infection and tissue damage following infection [15,16]. The ability to administer MAIT antigens intranasally may also overcome some issue of vaccine hesitancy, and a pan-vaccination approach could be a valuable tool in the event of future pandemics. The relevance of MAIT cells in all infectious disease contexts remains unknown, especially since many bacterial pathogens do not produce riboflavin-derived metabolites. However, the activation of MAIT cells in a bystander fashion indicates that this cell type plays a role in many infectious diseases, as has been illustrated in several of the studies noted herein. Further research into the role of MAIT cells in infectious disease will help to illuminate this. Notwithstanding, several hurdles remain before the MR1-MAIT axis can be effectively used for vaccine development.

Given that MAIT cells rapidly produce inflammatory cytokines (e.g., IFN-γ, TNF, IL-17) on activation, there is a risk that MR1-targeted interventions could provoke excessive local inflammation or tissue damage. This is a particular concern for mucosal delivery, where inflammation can be detrimental to barrier and physiological functions of the lung. Moreover, formulations that incorporate TLR ligands or other strong adjuvants may amplify inflammatory responses. For any novel MAIT-direct adjuvant or therapeutic, careful preclinical safety profiling is essential.

Another important question to address is whether those promising results observed in murine studies when using 5-OP-RU (with or without another MAIT cell co-stimulator) can be translated to human application. While promising MR1-mediated MAIT cell therapies have been observed in several murine models of infectious disease, including for *F. tularensis* [24], *L. longbeachae* [24,42], *K. pneumoniae* [43], and influenza [46], preliminary data in NHP models suggests that the addition of 5-OP-RU to healthy macaques only has a limited ability to expand MAIT cells [48]. Moreover, in NHP studies, loss of MAIT cell function was observed upon treatment with 5-OP-RU, and blocking the expression of the T cell exhaustion marker PD-1 did not affect MAIT cell expansion and only partially restored MAIT cell function [48].

MAIT cells play a key role in protecting and maintaining the integrity of the mucosal barrier and 5-OP-RU is a metabolite that is naturally found in the mucosa [15,16,31]. Accordingly, the body is frequently exposed to, and is exquisitely sensitive to, nanomolar concentrations of 5-OP-RU. This contrasts with mice who naturally have low numbers of MAIT cells, and who have MAIT cells of both the MAIT-1 and -17 phenotypes [15]. Moreover, most laboratory mice are housed in a specific pathogen free environment which limits the development and expansion of MAIT cells in the thymus and their subsequent maturation to peripheral tissue [17]. Insofar, the lack of proliferation and downregulation of cytokine producing abilities that has been observed after 5-OP-RU antigenic challenge in NHP models may not represent a defect but may be a function of normal MAIT cell biology that exists to prevent immunopathology. This then begs the question: can MR1-mediated therapies be effective in the context of human disease?

The answer to this likely lies in the choice of MR1 ligand. Indeed, the loss of cytokine production after the 5-OP-RU treatment of *M. tuberculosis*-infected animals was only observed when cells were restimulated with 5-OP-RU, and not with PMA/ionomycin restimulation [48]. Whether restimulation with a MR1 ligand other than 5-OP-RU can be used to enhance MAIT cell proliferation and activation in vivo remains to be determined. It could also be that although the dose of 5-OP-RU used in the NHP studies is often in the nanomolar range, this may still be too high for the exquisitely sensitive surveillance mechanism of the NHP MAIT cell. Insofar, a MR1-depedent ligand other than 5-OP-RU may lead to better outcomes. Despite having an evolutionary conserved TCR, MAIT cells also exhibit some specificity in their ability to recognise ligands with the MAIT TCRβ chain being able to “fine-tune” responsiveness to certain ligands [66,67]. Accordingly, studies aimed at understanding how the structure of TCR-dependent MAIT ligands influence MAIT cell expansion and whether specific MAIT cell clonotypes respond differently to different MR1-presented antigens, both within the context of murine and NHP studies, are needed.

It may also be the case that MAIT cell exhaustion, dysfunction, or even undesired cytokine production can be overcome by T-cell or B-cell-mediated MAIT cell co-stimulation. For example, 5-OP-RU has been shown to synergise with viral vaccines to lead to better MAIT-cell-mediated protection against influenza [56]; however, when 5-OP-RU was added in the absence of a viral vaccine, no mice survived following influenza challenge [56]. In an alternative study using HA as an antigen, both groups of mice (i.e., HA antigen + 5-A-RU/MG or 5-A-RU/MG only), survived subsequent influenza challenge yet the mice gained weight faster when using the combination of HA + 5-A-RU/MG [46]. Again, this points to the added benefit of MAIT cell co-stimulation on vaccination outcome. Moreover, it should be noted that the administration of 5-OP-RU within the context of a viral vaccine platform did not render the MAIT cells anergic [56]. This result is encouraging and suggests that prime-boost immunisation protocols may be possible, although it remains to be seen whether a similar finding will be observed when using NHP models of infection.

## 7. Conclusions

There is much still to be learnt about the potential of TCR-dependent MAIT cell activation to improve vaccine efficacy for infectious diseases. Several murine studies pertaining to the ability of 5-OP-RU to provide protection against both bacterial and viral infection have been demonstrated. However, given the difference in MAIT phenotype and number between humans and mice, and the fact that murine studies are typically performed using specific pathogen-free mice, it remains to be determined whether the data from the murine studies can be replicated in NHP models. In addition, the MAIT cell research field could greatly benefit from access to TCR-dependent MAIT cell agonists other than 5-OP-RU. 5-OP-RU is not only unstable, which makes it difficult to administer, but it is a ligand that the human MAIT cell is routinely exposed to. This may make it too potent an antigen for use in a vaccination setting, particularly one where only the MAIT agonist is added. The effect of MAIT co-stimulation via the addition of protein antigen(s) + 5-OP-RU has not been tested in NHP models of vaccination against infectious disease and may prove to be effective. However, this remains to be determined, as does the ability of other MR1 ligands to improve long-term immunity within the context of more traditional vaccine formulations for infectious disease.

## Figures and Tables

**Table 2:** MR1 Antigen + protein or carbohydrate antigen.

MR1 Ligand/ Antigen	Disease Model	MAIT Cell Expansion	MAIT Cell Phenotype	Mechanistic Insight	Challenge	Reference
5-A-RU/MG + OVA	Model antigen (OVA)	_	CD40L ↑, CD69 ↑ PD1 ↑, CD25 ↑	MAIT (MR1)-DC signalling Priming T_FH_ cells/GC B cells, IgG1 ↑, IgA ↑	_	Pankhurst et al. 2023 [46]
Ribityl lumazine derivative + OVA	Model antigen (OVA)	_	_	Total IgG ↑, IgG1 ↑, IgG2a ↑, IgM ↑	_	Takasaki et al. 2024 [54]
5-A-RU/MG + Pam_2_ + *V. cholerae O1* (live)	Cholera	Yes	CD69 no change	Antibody and B cell responses: no significant effect	_	Jensen et al. 2022 [55]
5-A-RU + MG + *V. cholerae O1* polysaccharide conjugate	Cholera	Yes	_	IgG ↑ (BAL only) Correlated to higher BAL MAIT frequency	_	Jensen et al. 2022 [55]
5-OP-RU + influenza vaccines (live attenuated)	Influenza	Yes In situ expansion	CD69 ↑, Ki67 ↑ MAIT1	TLR-3 dependent IFN-γ dependent CD8^+^ T cell ↑ virus specific response	75% survival (otherwise lethal)	Rashu et al. 2023 [56]
5-A-RU + MG + HA (subunit vaccine)	Influenza	See 5-OP-RU + OVA studies	_	IgG ↑, IgG1 ↑ IgG2b ↑, IgA ↑	100% protection against X31 challenge	Pankhurst et al. 2023 [46]
5-OP-RU + spike protein (viral vector)	SARS-CoV-2	Yes	_	_	_	Rashu et al. 2023 [56]
5-A-RU + MG + spike protein (subunit vaccine)	SARS-CoV-2	See 5-OP-RU + OVA studies	_	IgG ↑, IgG1 ↑, IgG2b ↑ IgA ↑, Antibodies had spike protein neutralising capacity	_	Pankhurst et al. 2023 [46]

## Data Availability

Not applicable.

## Abbreviations

The following abbreviations are used in this manuscript:

5-A-RU	5-amino-6-d-ribitylaminouracil
5-OE-RU	5-(2-oxoethylideneamino)-6-d-ribitylaminouracil
APC	Antigen-presenting cell
BAL	Bronchoalveolar lavage
BCG	Bacillus Calmette–Guérin
CCR2	C–C chemokine receptor type 2
CFU	Colony-forming unit
CpG	Cytosine–phosphate–guanine (CpG oligonucleotide)
DC	Dendritic cell
DMSO	Dimethyl sulfoxide
dpi	Days post infection
dsRNA	Double-stranded RNA
EC_50_	Half-maximal effective concentration
GC	Germinal centre
GM-CSF	Granulocyte–macrophage colony-stimulating factor
HA	Hemagglutinin
IAV	Influenza A virus
ICOS	Inducible T-cell co-stimulator
IFN	Interferon
IFNAR	Interferon-α/β receptor (type I interferon receptor)
Ig	Immunoglobulin
IL	Interleukin
Ki-67	Marker of proliferation Ki-67
LPS	Lipopolysaccharide
Ly6C	Lymphocyte antigen 6 complex, locus C
MAIT	Mucosal-associated invariant T (cell)
MG	Methylglyoxal
MHC	Major histocompatibility complex
mLN	Mediastinal lymph node
MR1	MHC class I-related protein 1
NHP	Non-human primate
NK	Natural killer (cell)
NOS2	Nitric oxide synthase 2
OVA	Ovalbumin
Pam_2_CSK_4_	Pam_2_Cys-Ser-(Lys)_4_ (synthetic TLR2/6 agonist)
Pam_2_Cys	*S*-[2,3-bis(palmitoyloxy)propyl]-l-cysteine
PAMP	Pathogen-associated molecular pattern
PBS	Phosphate-buffered saline
PD-1	Programmed cell death protein 1
polyI:C	Polyinosinic–polycytidylic acid
RORγt	Retinoic acid receptor-related orphan receptor gamma t
rVSV	Recombinant vesicular stomatitis virus
SARS-CoV-2	Severe acute respiratory syndrome coronavirus 2
TB	Tuberculosis
T-bet	T-box expressed in T cells (TBX21)
TCR	T cell receptor
T_FH_	T follicular helper (cell)
Th	T helper (cell)
TLR	Toll-like receptor
TNF	Tumour necrosis factor
